# Supplementary material for: DMRforPairs: identifying Differentially Methylated Regions between unique samples using array based methylation profiles
Source: BMC Bioinformatics. 2014 May 15;15:141. doi: 10.1186/1471-2105-15-141 (PMC4046028; doi:10.1186/1471-2105-15-141)
Supplement: Additional file 2 — DMRforPairs output for the comparison of A431-MCF7 and NA17018-NA17105. Please start from the HTML files in each folder. Available via the BMC Bioinformatics website. [file 1471-2105-15-141-S2.zip › 1394847754114233_MOESM2_ESM/A431_MCF7/figures/39582.pdf]

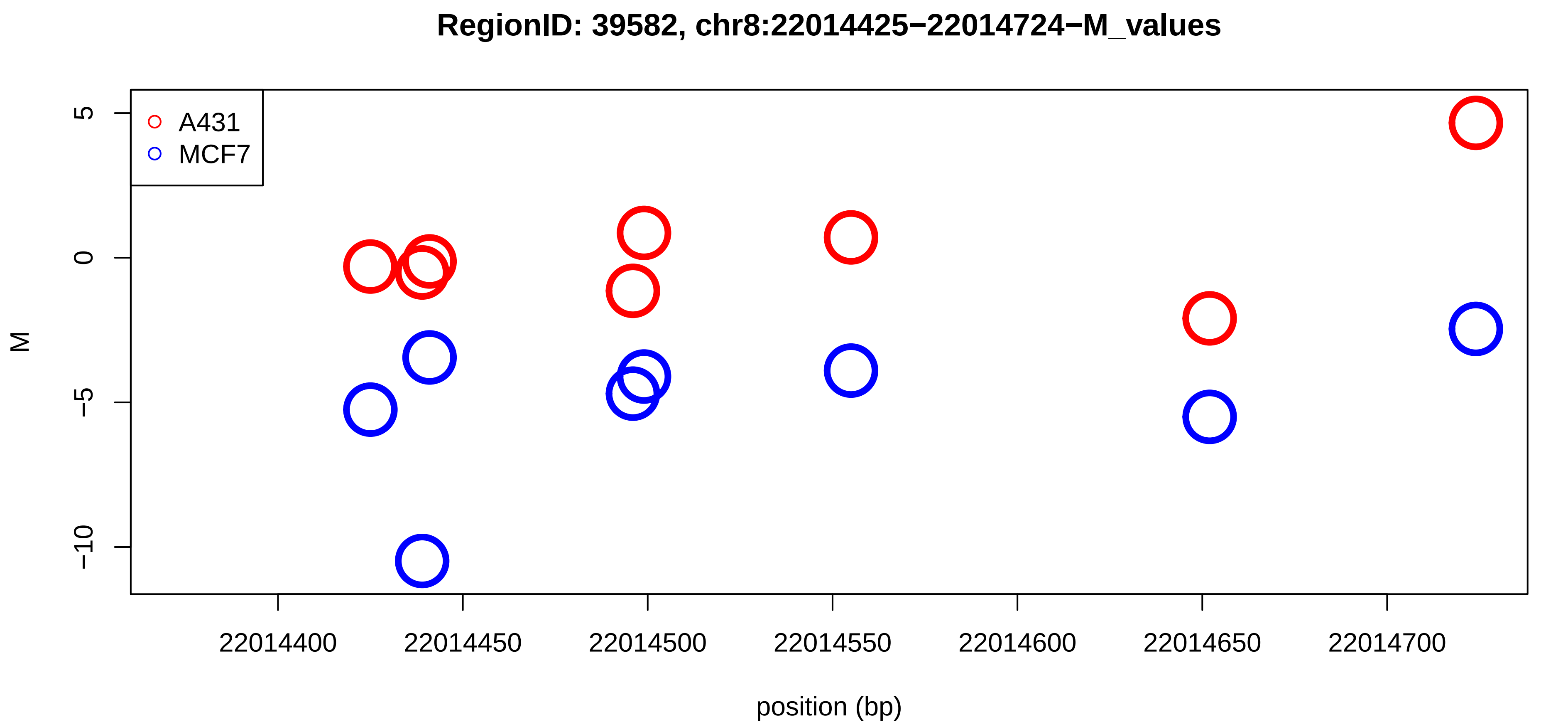
RegionID: 39582, chr8:22014425−22014724−M_values
A431
MCF7
M
5
0
−5
−10
22014400
22014450
22014500
22014550
22014600
22014650
22014700
position (bp)

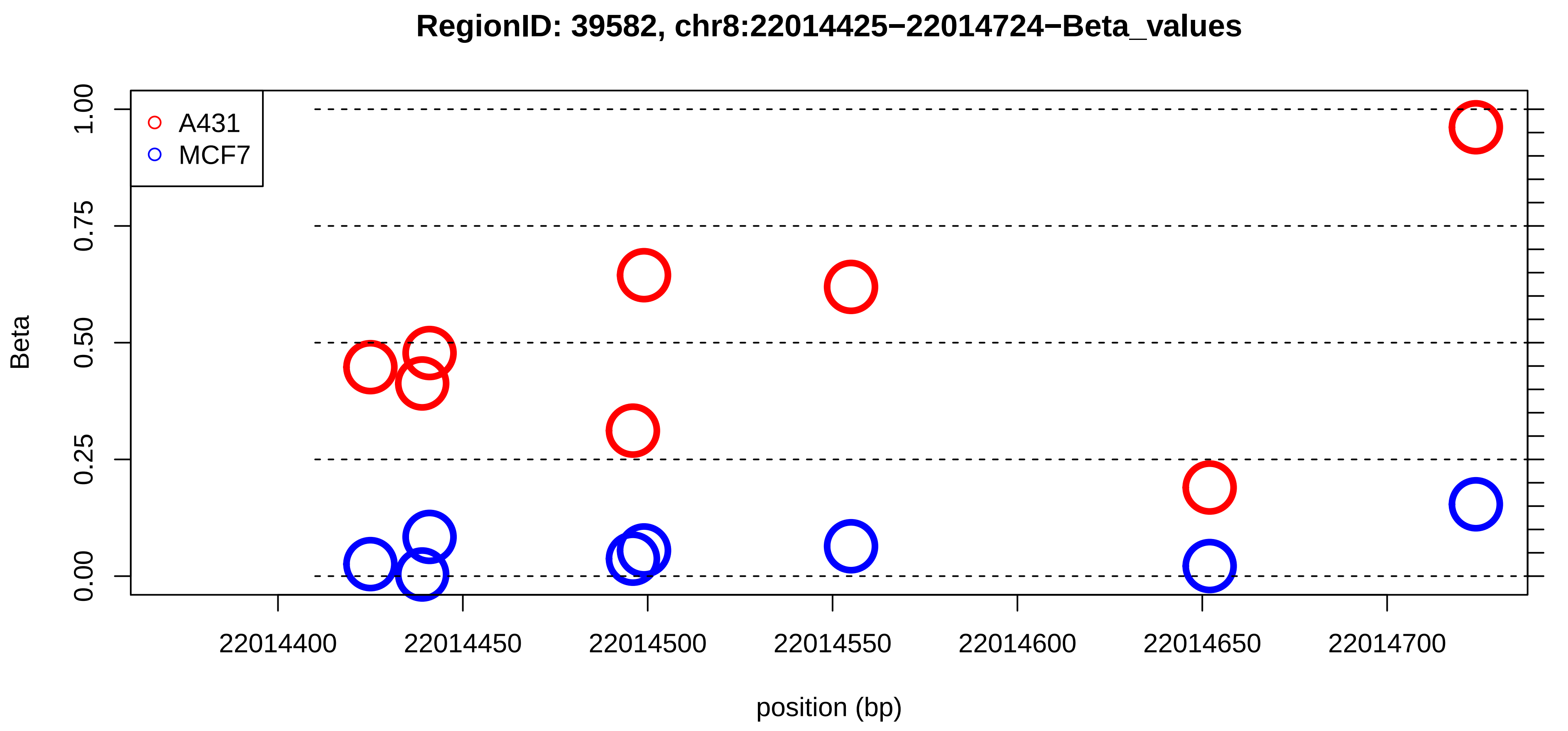
RegionID: 39582, chr8:22014425−22014724−Beta_values
A431
MCF7
Beta
1.00
0.75
0.50
0.25
0.00
22014400
22014450
22014500
22014550
22014600
22014650
22014700
position (bp)

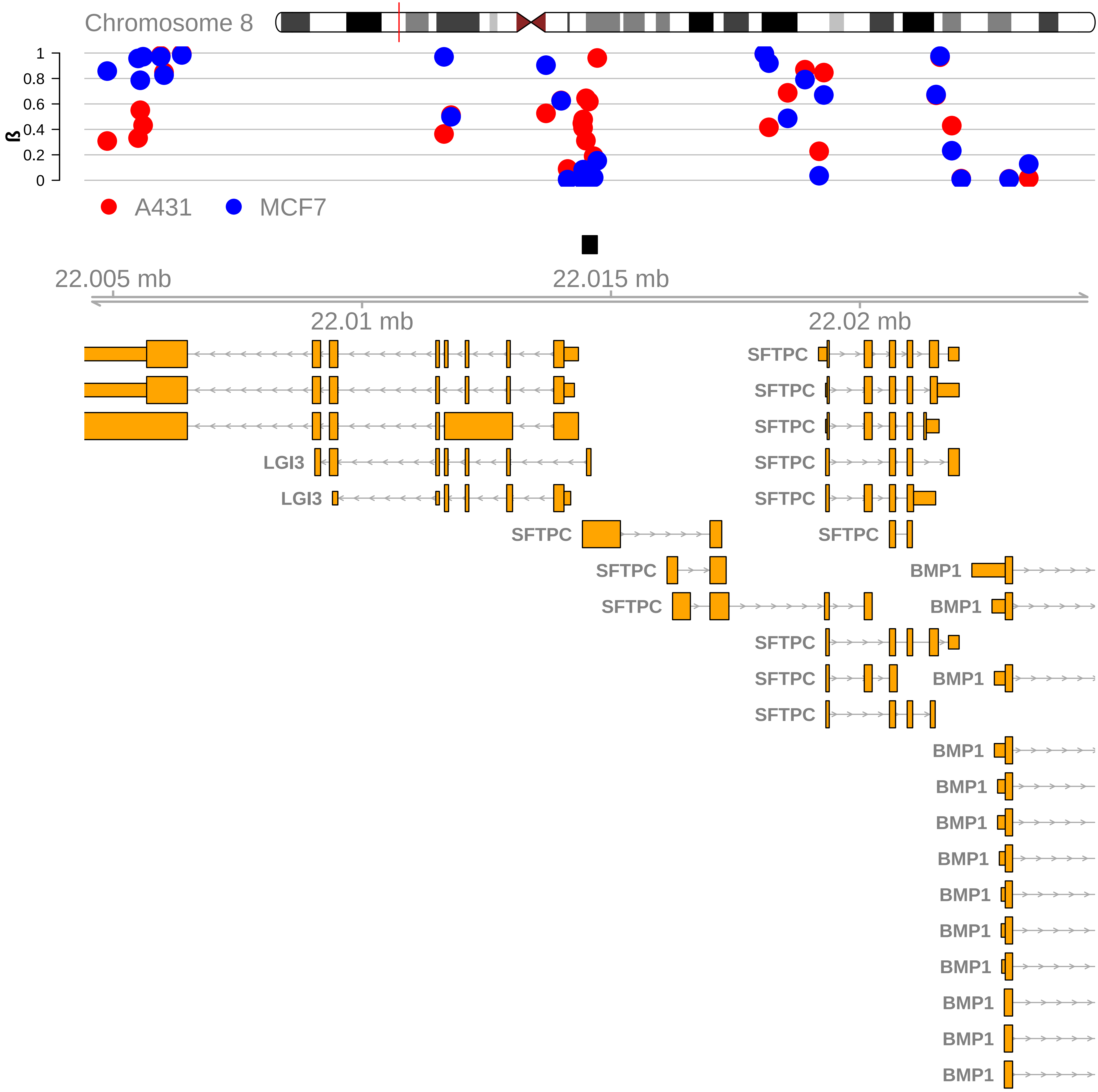

Chromosome 8
ß
1
0.8
0.6
0.4
0.2
0
A431
MCF7
22.005 mb
22.015 mb
22.01 mb
22.02 mb
SFTPC
SFTPC
SFTPC
LGI3
SFTPC
LGI3
SFTPC
SFTPC
SFTPC
SFTPC
BMP1
SFTPC
BMP1
SFTPC
SFTPC
BMP1
SFTPC
BMP1
BMP1
BMP1
BMP1
BMP1
BMP1
BMP1
BMP1
BMP1
BMP1
